# The scientific evidence of commercial AI products for MRI acceleration: a systematic review

**DOI:** 10.1007/s00330-025-11423-5

**Published:** 2025-02-19

**Authors:** Stefan J. Fransen, Christian Roest, Frank F. J. Simonis, Derya Yakar, Thomas C. Kwee

**Affiliations:** 1 Department of Radiology, University Medical Center Groningen, Groningen, The Netherlands; 2 TechMed Centre, Technical University Twente, Enschede, The Netherlands

**Keywords:** Magnetic resonance imaging, Acceleration, Artificial intelligence, Radiology, Evidence-based practice

## Abstract

**Objectives:**

This study explores the methods employed by commercially available AI products to accelerate MRI protocols and investigates the strength of their diagnostic image quality assessment.

**Materials and methods:**

All commercial AI products for MRI acceleration were identified from the exhibitors presented at the RSNA 2023 and ECR 2024 annual meetings. Peer-reviewed scientific articles describing validation of clinical performance were searched for each product. Information was extracted regarding the MRI acceleration technique, achieved acceleration, diagnostic performance metrics, test cohort, and hallucinatory artifacts. The strength of the diagnostic image quality was assessed using scientific evidence levels ranging from “product’s technical feasibility for clinical purposes” to “product’s economic impact on society”.

**Results:**

Out of 1046 companies, 14 products of 14 companies were included. No scientific articles were found for four products (29%). For the remaining ten products (71%), 21 articles were retrieved. Four acceleration methods were identified: noise reduction, raw data reconstruction, personalized scanning protocols, and synthetic image generation. Only a limited number of articles prospectively demonstrated impact on patient outcomes (*n* = 4, 19%), and no articles discussed an evaluation in a prospective cohort of > 100 patients or performed an economic analysis. None of the articles performed an analysis of hallucinatory artifacts.

**Conclusion:**

Currently, commercially available AI products for MRI acceleration can be categorized into four main methods. The acceleration methods lack prospective scientific evidence on clinical performance in large cohorts and economic analysis, which would help to get a better insight into their diagnostic performance and enable safe and effective clinical implementation.

**Key Points:**

***Question*** *There is a growing interest in AI products that reduce MRI scan time, but an overview of these methods and their scientific evidence is missing*.

***Findings*** *Only a limited number of articles (n* *=* *4, 19%) prospectively demonstrated the impact of the software for accelerating MRI on diagnostic performance metrics*.

***Clinical relevance*** *Although various commercially available products shorten MRI acquisition time, more studies in large cohorts are needed to get a better insight into the diagnostic performance of AI-constructed MRI*.

## Introduction

Medical imaging plays a central role in patient care as a method for diagnosis, treatment planning, and monitoring. Increased accessibility to medical imaging is estimated to avert almost 2.5 million deaths caused by cancer [1]. Among the various imaging techniques, MRI is the crucial imaging method for cancers such as brain, prostate, and rectal cancer [2, 3]. However, increasing the accessibility of MRI requires overcoming several challenges: the high costs of installation, operation, and maintenance, as well as the long acquisition times that limit patient throughput [4].

Multiple strategies have been reported in scientific literature to shorten the acquisition times of MRI while preserving the diagnostic quality [5, 6]. A widely applied conventional MRI acceleration technique, called parallel imaging, reduces the acquisition of raw MRI data and restores the missing information during image reconstruction using calibration scans, which are part of the same acquisition. However, the acceleration factor that can be achieved using conventional techniques is relatively low compared to novel techniques based on artificial intelligence (AI) enhanced acceleration [6–9]. AI-enhanced techniques can increase acceleration factors by learning to restore missing information based on information from large datasets, offering greater potential for reducing acquisition times.

To safely integrate AI algorithms for MRI acquisition acceleration into clinical practice, it is crucial to understand how these algorithms have been clinically validated and to identify potential biases that might affect their outcome on patients [10]. While scientific evaluation of AI products intended for clinical use is crucial for transparent and trustworthy AI, scientific evidence of commercially available AI software for use in radiology is often lacking [11]. Scientific evidence is especially important in the novel field of AI-based MRI reconstruction, as reconstruction hallucinations might be introduced, potentially affecting the diagnosis [12, 13]. Furthermore, visual image evaluation, the most commonly used evaluation metric, does not necessarily translate to diagnostic patient outcome performance [13]. Despite the growing interest in and availability of MRI acceleration products, an overview of the scientific evidence for commercially available AI methods for MRI acceleration is lacking.

This study systematically explores the methods employed by commercially available AI products to accelerate MRI protocols and investigates the strength of their diagnostic image quality assessment. An overview of the available AI methods for MRI acceleration and their diagnostic image quality assessment is provided.

## Materials and methods

### Article selection

This study included articles describing the validation of AI products to shorten the MRI acquisition time which were exhibited at the Radiological Society of North America (RSNA) 2023 and European Congress of Radiology (ECR) 2024 annual meetings. These conferences are the largest radiology events in both America and Europe, making them ideal to assess the advancements in AI for MRI acceleration. All exhibitors were searched using the Google search engine, and products were selected based on the companies’ web pages. Commercial products were deemed eligible for inclusion if they were explicitly designed to shorten MRI acquisition time with AI. We defined commercial availability as being marketed on the companies’ web pages. Exclusion criteria were (1) products not using AI or (2) products focused on enhancing patient throughput without reducing scan time (e.g., MRI patient planning products).

Two reviewers (S.J.F., with eight years of experience in medical imaging and three years of experience in AI; C.R., with four years of experience in medical imaging and nine years of experience in AI) searched company websites and Google Scholar to identify peer-reviewed scientific articles on each product. Differences were resolved by mutual agreement. On the company websites, listings of publications were searched. On Google Scholar, the search term “[product] MRI acceleration” was used. Up to three of the most relevant articles were included for each product, based on an initial scientific evidence assessment (e.g., prospective studies, larger sample sizes, and clinical relevance) after abstract reading. Only peer-reviewed, original articles written in English were included if they aimed to assess the image quality after AI-based MRI reconstruction. Letters, commentaries, reviews, study protocols, white papers, and case reports were excluded. The search was updated until 5th June 2024; articles published after this date were not included.

### Diagnostic image quality assessment

Our diagnostic image quality assessment focused on the clinical relevance (i.e., how easy it can be used in the clinic) of the reported outcome measure in the reconstructed image evaluation. A commonly used method to evaluate reconstructed image quality is to compare the standard and accelerated AI protocols in a reader study. Although this comparison cannot attribute the acceleration solely to the consolidated effect of DL reconstruction (i.e., other advanced reconstruction techniques might give similar results on undersampled data), these reader studies must consider the workload for its readers and are well suited for assessing image quality in accelerated MRI protocols. These reader studies can be performed in various ways that affect the strength of the clinical relevance of the employed analysis. Therefore, we developed a framework to categorize the different strengths of diagnostic image quality assessment methods into scientific evidence levels.

The developed framework (Table 1) was primarily based on previously published frameworks to assess the scientific evidence of medical AI products [11, 14]. We made changes using QUADAS-2 guidelines to make the framework suitable for the diagnostic quality assessment of AI-reconstructed accelerated MRI images [15]. Visual and diagnostic assessments were classified separately since diagnostic quality might still be reduced in visually appealing images [13]. Visual metrics compare the images on similarity and assess the image quality (levels 1 and 2). Diagnostic metrics compare the images on their diagnostic value and assess the patient outcome (levels 3–6). Furthermore, a separate classification was made between retrospective (levels 3 and 4) and prospective test cohorts (levels 5 and 6). At last, we separately classified prospective studies with a power analysis (level 5a) from prospective studies without a power analysis (level 5b). In line with the QUADAS-2 guidelines [15], the evidence levels were merged into three classifications that indicate the strength of the clinical relevance of the reported method to evaluate the reconstructed image quality: levels 5 and 6 were interpreted as a high evidence level, levels 3 and 4 as medium evidence level, and levels 1 and 2 as low evidence level.

**Table 1:** Scientific evidence assessment framework adapted for studies aimed to assess the diagnostic image quality of AI-based accelerated MRI images

Level	Explanation	Examples
Level 1	Potential technical efficacy The article demonstrates the technical feasibility of the product for clinical purposes.	Simulated data with visual assessment
Level 2	Technical efficacy The article demonstrates the feasibility of the product to be clinically applied.	Real data with visual assessment
Level 3	Potential diagnostic efficacy The article demonstrates the potential added clinical value.	Diagnostic biomarker assessment (e.g., diagnostic confidence)
Level 4	Diagnostic efficacy The article retrospectively demonstrates the potential impact of the software on diagnostic performance metrics.	Diagnostic assessment (e.g., sensitivity) on retrospective data
Level 5a	Patient outcome efficacy The article prospectively demonstrates the impact of the software on diagnostic performance metrics.	Diagnostic assessment (e.g., sensitivity) on prospective data without a power analysis
Level 5b	Generalizable patient outcome efficacy The article prospectively demonstrates the impact of the software on diagnostic performance metrics.	Diagnostic assessment (e.g., sensitivity) on prospective data with a power analysis
Level 6	Societal efficacy The article demonstrates the impact of the software on society by performing an economic analysis.	Evaluation of added value in terms of prospective determined costs per quality-adjusted life year

### Analysis

From the included articles, clinical information was retrieved about the targeted body part, test cohort, and diagnostic performance metrics (e.g., visual or diagnostic evaluation). The diagnostic performance metrics were categorized into quantitative metrics (i.e., objectively calculated metrics), subjective metrics (i.e., perceived visual quality metrics), and diagnostic performance metrics (i.e., comparing test results to the patient’s true disease status as assessed by a reference standard). In addition, technical information about the method of MRI acceleration was retrieved (i.e., hallucinatory artifacts and model architecture). The acceleration factor is defined as the amount of time reduction compared to the original protocol.

In the analysis, articles were subcategorized according to their specific method to shorten MRI acquisition time. Within each different subcategory, the evidence level of the diagnostic image quality assessment was determined based on the extracted information and the scientific evidence framework.

## Results

### Article selection

**Fig. 1:**
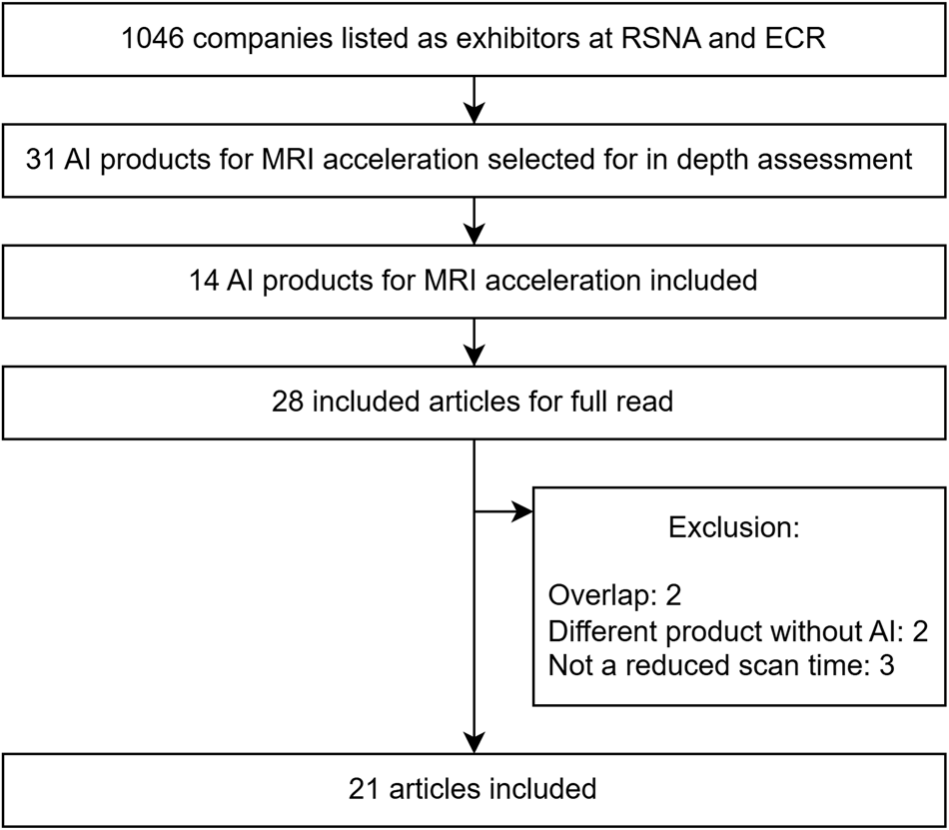
STARD diagram of article selection

A total of 1046 companies were initially included, of which 805 were RSNA exhibitors and 241 were ECR exhibitors. After preselection by reading the companies’ webpages, 31 products were selected. After an in-depth webpage search, the final inclusion contained 14 products from 14 companies. After searching the companies’ webpages and Google Scholar for peer-reviewed research articles, 28 articles were identified for 10 products. For four products, no articles describing clinical performance validation were found. After fully reading the articles, seven were excluded: two because of overlap, two because the product in the manuscript did not use AI, and three because the product was not tested to reduce scan time. A total of 21 articles were included in this study. Figure 1 provides a STARD diagram of the article selection.

### Acceleration techniques

We categorized the acceleration techniques into four groups based on the specific method employed to accelerate the MRI acquisition: noise reduction, raw data reconstruction, synthetic image generation, and personalized scanning protocol. These categories were applied to classify and analyze the techniques discussed in our study. Table 2 provides an overview of the extracted information from the included articles, including body part, type of cohort, diagnostic performance metrics, and evidence level. The main results from Table 2 are described in the following sections.

**Table 2:** Scientific evidence on diagnostic image quality assessment results




The colors represent the scientific evidence level classifications: levels 5 and 6 were interpreted as a high evidence level and shown in green, levels 3 and 4 as a medium evidence level and shown in yellow, and levels 1 and 2 as a low evidence level and shown in red. Accel. indicates the acceleration factor calculated as the amount of time reduction compared to the original protocol, NRMSE indicates the normalized root mean squared error, PSNR indicates the peak signal-to-noise ratio, RMSE indicates the root mean square error, RMSPE indicates the root mean square percentage error, SSIM indicates the structural similarity index measure

### Noise reduction

#### Scientific evidence

**Fig. 2:**
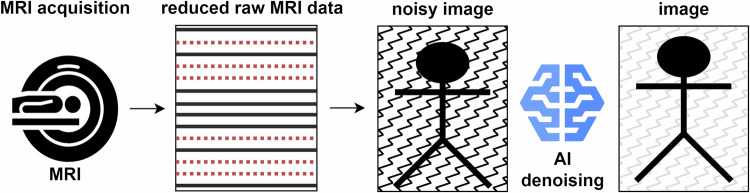
Schematic overview of an example of the noise reduction method for MRI acceleration. The raw MRI data acquisition is reduced and reconstructed into a noisy image using regular reconstruction. An AI-denoising model is employed to produce an image with good diagnostic quality

Noise reduction shortens MRI scan time by acquiring images with a lowered signal-to-noise ratio through raw MRI undersampling and applying post-acquisition image enhancement using denoising AI. Figure 2 provides a schematic overview of the general noise reduction method for MRI acceleration. No peer-reviewed scientific evidence was found for the two products (GOPView MRI2PLUS, Context vision; Echolon Synergy MRI, FUJIFILM Healthcare). For four products (SwiftMR, AIRS Medical; Intelligent Quick Magnetic Resonance, Medic Vision Imaging Solutions; SubtleMR, Subtle Medical; SubtleMR, Incepto Medical; AiCE, Canon Medical Systems), a total of eight peer-reviewed articles were included addressing noise reduction. Of the included studies, three studies had high evidence [16–18], four studies had medium evidence [19–22], and one study had low evidence [23], as shown in Table 2. One of the high-evidence studies performed a diagnostic assessment on prospective data with a power analysis [18]. The reported MRI acceleration of all papers with noise reduction was between 38% and 86%.

#### Diagnostic performance metrics

The highest acceleration, 86%, was reported by Matsuyama et al in a study that compared accelerated AI-denoised with unaccelerated pancreas MRI scans. In their analysis, the authors reported quantitative visual, subjective visual, and diagnostic performance metrics. They showed that denoising AI after MRI acceleration is useful for improving visual image quality metrics and intraductal papillary mucinous neoplasm detection (i.e., diagnostic quality metric), as evidenced by all metrics. This uniformity of diagnostic and visual performance metric outcomes was not observed in all studies, with Lee et al showing a difference in outcome between quantitative visual and subjective visual metrics [16] and Yao et al showing a difference in diagnostic performance for specific pathologies in the same knee MRI [18], emphasizing the importance of extensive diagnostic assessment alongside visual metrics.

#### Technical specifications

The technical specifications of the denoising AI were comparable between products, leveraging U-Net-based models to reconstruct high-quality images from low-quality inputs. A risk of AI-denoising is smoothing the image, which may remove diagnostic-relevant pathologies. This effect was discussed in four articles (*n* = 4, 50%) [16, 19–21]. Yao et al proposed a solution for this problem by focusing on the noise map during training to ensure no diagnostic information is removed from the image [18].

### Raw data reconstruction

#### Scientific evidence

**Fig. 3:**
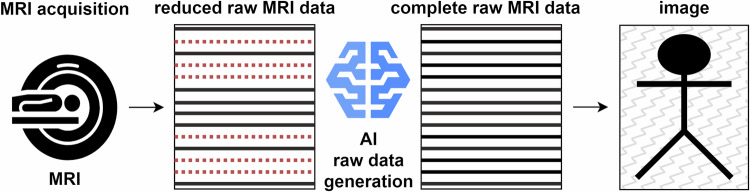
Schematic overview of raw data reconstruction method for MRI acceleration. An image is acquired with reduced raw MRI data and completed using an AI raw data generation model. After the raw MRI data is predicted, the image is reconstructed using regular reconstruction

Raw data reconstruction shortens MRI scan time by acquiring less raw MRI data, similar to the noise reduction method, and using an AI algorithm to predict the missing raw MRI data. Figure 3 provides a schematic overview of the method of raw data generation for MRI acceleration. Five products (SwiftMR, AIRS Medical; SmartSpeed, Philips Healthcare; Deep Resolve, Siemens Healthineers; AIR Recon DL, GE Healthcare; uMR780 and uMR790, United Imaging) with 12 evaluation studies were included, as shown in Table 2. Of the included studies, one study had high evidence [24], ten had medium evidence [25–33], and one had low evidence [34], as shown in Table 2. The reported MRI acceleration with raw data reconstruction was between 19% and 96%.

#### Diagnostic performance metrics

The highest acceleration of 96% was demonstrated by Yan et al [31] in cardiac MRI, where the authors reportedly reduced the scan time from 176 s to 6.7 s [35]. Their results showed similar diagnostic performance between the standard and accelerated MRI scans but slightly reduced subjective image quality. No articles on raw data reconstruction prospectively demonstrated the impact of the software on patient outcomes (level 5). Multiple articles included prospective scans, but these were either acquired in volunteers or the diagnostic assessment only involved diagnostic biomarkers (e.g., delineation and clarity of anatomical structures or anatomical functionality without diagnostic predictions).

#### Technical specifications

The technical specifications of the AI raw data generation were comparable between products: leveraging U-Net-based models to reconstruct an image from raw MRI data. In one study, AI-based MRI reconstruction was applied to cardiac imaging for which a recurrent neural network was employed that takes the time component of the scan into account [35]. Furthermore, two studies described simulated raw data usage [25, 35]. Caution should be paid when results from simulated raw data are applied to real data, which may result in different performances (i.e., biased performance arising from naïve, faulty data processing) [36]. Finally, the risk of AI raw data generation is the generation of hallucinatory artifacts that may result in false-positive or false-negative lesions, as discussed by four articles (*n* = 4, 33%) [25, 27, 28, 32].

### Synthetic image generation

#### Scientific evidence

**Fig. 4:**
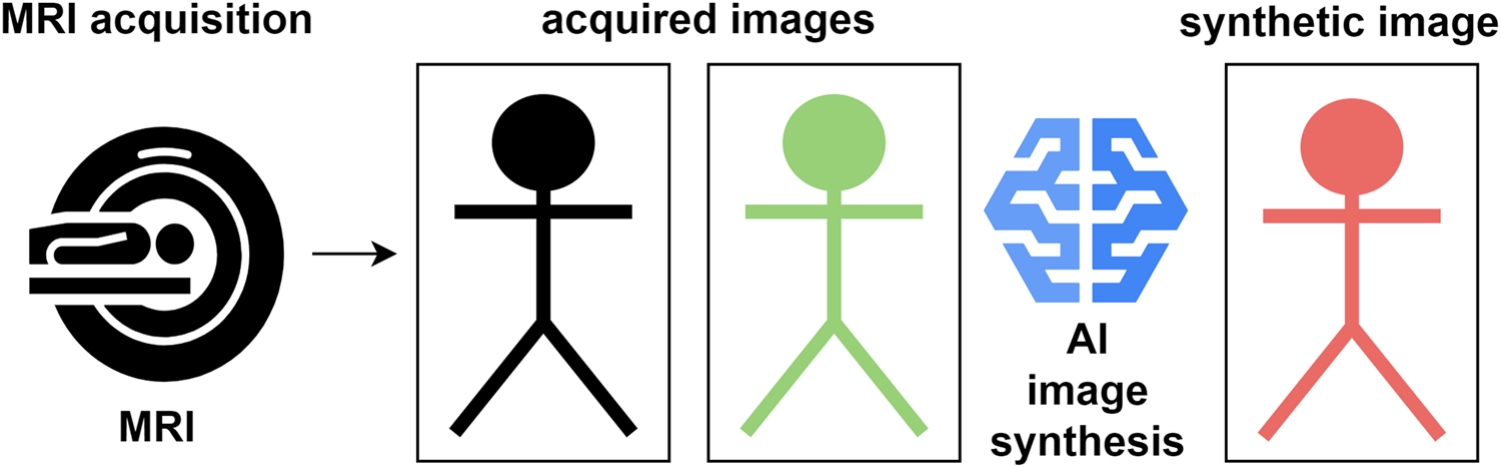
Schematic overview of the synthetic image generation method for MRI acceleration. First, a couple of MRI images are acquired separately. These are inputs for AI to synthesize other images with different MRI contrasts

Synthetic image generation shortens MRI scan time by reducing repeated measurements and creating synthetic images with new contrasts from existing acquired images using AI image synthesis. Figure 4 provides a schematic overview of the synthetic image generation method. One product aimed to shorten the MRI scan time with AI image synthesis (SubtleSYNTH; Subtle Medical). One article was included with a medium evidence level, see Table 2. This article reported an MRI acceleration with raw data reconstruction of 25% [37].

#### Diagnostic performance metrics

The included paper on synthetic image generation compared synthesized to acquired short-T1 inversion recovery (STIR) images as a method to detect lumbar spine pathologies [37]. Their results showed a 3.23% decrease in agreement between radiologists in detecting spine pathologies and a 0.85% increase in agreement in categorizing disease severity when using the synthesized STIR image compared to the acquired STIR image (*p* < 0.05). The diagnostic assessment was supplemented with a visual quality analysis that showed an increase in visual quality of 0.5 on a 5-point Likert scale (*p* < 0.05). The decreased agreement in detecting spine pathologies would have been missed when only focusing on visual quality.

#### Technical specifications

The AI algorithm used in this product is a convolutional neural network with a focus on specific areas of interest within the image by incorporating an anatomy segmentation map and a pathology saliency map during training [37]. Moreover, a weighted combination of loss functions was used to optimize the synthetic generation: the L1 loss for absolute differences on a pixel-by-pixel basis, the structural similarity index measure loss for the structural similarity, and the Edge loss for structural details. Finally, a risk of AI image synthesis is hallucinatory artifacts, also discussed in the article [37].

### Personalized scanning protocol

#### Scientific evidence

**Fig. 5:**
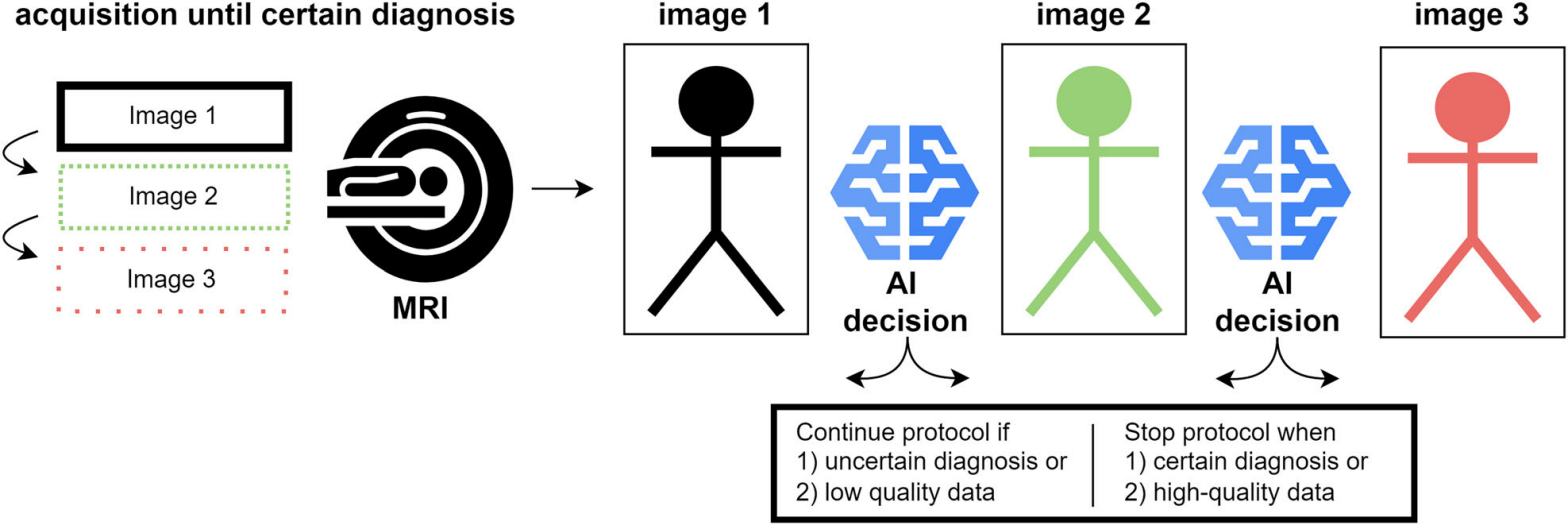
Schematic overview of personalized scanning method for MRI acceleration. The image acquisition continues until the AI decision model concludes that a certain diagnosis can be made. After each data acquisition step, the AI decision model predicts whether to continue or stop the MRI acquisition

A personalized scanning protocol shortens MRI scan time by omitting redundant steps in the protocol determined by a decision AI. Figure 5 provides a schematic overview of an example of a personalized scanning protocol method for MRI acceleration. Two products without peer-reviewed scientific evidence were included in the RSNA and ECR exhibitor lists. One product optimizes MRI acquisition by real-time monitoring data quality, allowing efficient scan durations to capture high-quality, motion-free data (One Click MRI, Vista.AI). The other product (Smart Protocol, Cerebriu) terminates the MRI acquisition when a sufficiently accurate diagnosis can be given using the abbreviated protocol.

## Discussion

To increase AI product transparency and contribute to trustworthy AI, this study explored the methods of commercially available AI products to accelerate MRI protocols and investigated the strength of their diagnostic image quality assessment. The current commercially available products to shorten the MRI acquisition time can be categorized into four methods: noise reduction, raw data reconstruction, synthetic image generation, and personalized scanning protocol. Most products were found for noise reduction (*n* = 6, 43%), which may reflect that this method is more accessible to implement, and AI can achieve good performance. A limited number of scientific articles have high evidence (level ≥ 5; *n* = 4, 19%), and no articles demonstrated the impact of the software on society by performing an economic analysis (level 6). Moreover, no articles were included with a prospective cohort of > 100 patients.

Due to the infrequent occurrence of hallucinatory artifacts that may be introduced with AI-based MRI acceleration methods, diagnostic studies in large cohorts are needed for thorough investigation. Only 9 (43%) of the included articles in our study mentioned the risk of hallucinatory artifacts, and none of the articles performed an analysis of hallucinatory artifacts. These hallucinations can be a problem in AI-reconstructed MRI, occurring infrequently but with potentially significant diagnostic implications. For example, reconstruction hallucinations can introduce false-positive lesions or remove significant lesions from the image [12, 13]. As previously mentioned, besides hallucinatory artifacts, diagnostic image quality assessment should focus on the choice of diagnostic quality metrics. Multiple studies showed a difference between diagnostic and visual performance metric outcomes [13, 16, 18]. Relying solely on visual diagnostic quality metrics or small cohorts is inadequate to fully understand the diagnostic performance and risk of hallucinatory artifacts of AI-reconstructed MRI, emphasizing the necessity of diagnostic studies in large cohorts.

When comparing our results with the current literature, one study on the scientific evidence of commercially available AI products emerges [11]. Although this study offers an extensive and ongoing overview of AI products (www.radiology.healthairegister.com), it was published in 2021 and did not include AI products for image reconstruction. Moreover, as a technique that has only recently seen commercialization, all of our included articles were published after their inclusion date of April 2020. When comparing the results, our study found a higher (52% vs 18%) percentage of products with scientific evidence for potential impact on diagnosis, patient outcome, or costs (level ≥ 4). This might indicate that the market has been maturing since 2020.

This study has limitations. The performed literature search was systematic and included both website screening and web searching of products presented at the RSNA 2023 and ECR 2024. Nevertheless, we might have missed products that were not presented at the largest radiology conferences. By abstract reading, up to three articles per product were selected with the highest scientific evidence. This selection gives a good overview of the field, but some articles might have been missed. We also might have missed articles that do not use the product’s name or are undisclosed by the vendor. Moreover, we defined commercially available as being marketed on the companies’ web pages and were not able to verify whether products were actually commercially available through certification. Also, there may have been additional validation data present for, e.g., regulatory purposes, but there is no public database in Europe providing that data. Furthermore, although we only included peer-reviewed, published articles, our analysis did not focus on the quality of the statistical methods. In addition, we did not perform extensive research into potential conflicts of interest authors might have in any of these studies, so these cannot be completely ruled out. Investigating publication bias could be an interesting avenue for future studies.

In conclusion, the current commercially available products to shorten the MRI acquisition time can be categorized into four methods: noise reduction, raw data reconstruction, synthetic image generation, and personalized scanning protocol. The acceleration methods lack prospective scientific evidence on clinical performance in large cohorts and economic analysis, which would help to get a better insight into their diagnostic performance and enable safe and effective clinical implementation.

## Abbreviations

AI: Artificial intelligence
ECR: European Congress of Radiology
RSNA: Radiological Society of North America
STIR: Short-T1 inversion recovery
